# Preliminary data revealing efficacy of *Streptococcus salivarius* K12 (SSK12) in Periodic Fever, Aphthous stomatitis, Pharyngitis, and cervical Adenitis (PFAPA) syndrome: A multicenter study from the AIDA Network PFAPA syndrome registry

**DOI:** 10.3389/fmed.2023.1105605

**Published:** 2023-02-16

**Authors:** Francesco La Torre, Jurgen Sota, Antonella Insalaco, Giovanni Conti, Emanuela Del Giudice, Riccardo Lubrano, Luciana Breda, Maria Cristina Maggio, Adele Civino, Violetta Mastrorilli, Roberta Loconte, Marco Francesco Natale, Camilla Celani, Mery Romeo, Serena Patroniti, Cristina Gentile, Antonio Vitale, Valeria Caggiano, Carla Gaggiano, Federico Diomeda, Marco Cattalini, Giuseppe Lopalco, Giacomo Emmi, Paola Parronchi, Stefano Gentileschi, Fabio Cardinale, Emma Aragona, Farhad Shahram, Achille Marino, Patrizia Barone, Carla Moscheo, Burcugul Ozkiziltas, Francesco Carubbi, Ohoud Alahmed, Ludovica Iezzi, Benson Ogunjimi, Angela Mauro, Maria Tarsia, Ayman Abdel-Monem Ahmed Mahmoud, Henrique Ayres Mayrink Giardini, Petros P. Sfikakis, Katerina Laskari, Ewa Więsik-Szewczyk, José Hernández-Rodríguez, Bruno Frediani, Verónica Gómez-Caverzaschi, Abdurrahman Tufan, Ibrahim A. Almaghlouth, Alberto Balistreri, Gaafar Ragab, Claudia Fabiani, Luca Cantarini, Donato Rigante

**Affiliations:** ^1^Department of Pediatrics, Pediatric Rheumatology Center, Giovanni XXIII Pediatric Hospital, University of Bari, Bari, Italy; ^2^Division of Rheumatology, Department of Medical Sciences, Surgery and Neurosciences, Research Center of Systemic Autoinflammatory Diseases and Behçet’s Disease Clinic, University of Siena, Siena, Italy; ^3^Ospedale Pediatrico Bambino Gesù, IRCCS (European Reference Network for Rare Immunodeficiency, Autoinflammatory and Autoimmune Diseases Center), Rome, Italy; ^4^Pediatric Nephrology and Rheumatology Unit, Azienda Ospedaliera Universitaria G. Martino, Messina, Italy; ^5^Pediatric and Neonatology Unit, Department of Maternal Infantile and Urological Sciences, Sapienza University of Rome, Polo Pontino, Latina, Italy; ^6^Pediatric Rheumatology Unit, S.S. Annunziata Hospital, Chieti, Italy; ^7^Department of Health Promotion, Mother and Child Care, Internal Medicine and Medical Specialties (PROMISE) “G. D’Alessandro”, University of Palermo, Palermo, Italy; ^8^Pediatric Rheumatology and Immunology Unit, Vito Fazzi Hospital, Lecce, Italy; ^9^Pediatric Clinic, University of Brescia and Spedali Civili di Brescia, Brescia, Italy; ^10^Rheumatology Unit, Department of Emergency and Organ Transplantation, University of Bari, Bari, Italy; ^11^Department of Experimental and Clinical Medicine, University of Florence, Florence, Italy; ^12^Centre for Inflammatory Diseases, Department of Medicine, Monash Medical Centre, Monash University, Clayton, VIC, Australia; ^13^Division of Gastroenterology, Ospedali Riuniti Villa Sofia-Vincenzo Cervello, Palermo, Italy; ^14^Behcet’s Disease Unit, Rheumatology Research Center, Shariati Hospital, Tehran University of Medical Sciences, Tehran, Iran; ^15^Unit of Pediatric Rheumatology, ASST Gaetano Pini-CTO, Milan, Italy; ^16^Department of Clinical and Experimental Medicine, University of Catania, Catania, Italy; ^17^Pediatric Unit, Azienda Ospedaliero-Universitaria Policlinico “Rodolico-San Marco”, Catania, Italy; ^18^Division of Rheumatology, Department of Internal Medicine, Gazi University Faculty of Medicine, Ankara, Turkey; ^19^Department of Life, Health & Environmental Sciences and Internal Medicine and Nephrology Unit, Department of Medicine, University of L’Aquila and ASL Avezzano-Sulmona-L’Aquila, San Salvatore Hospital, L’Aquila, Italy; ^20^Pediatric Rheumatology, Department of Pediatrics, College of Medicine, King Saud University, Riyadh, Saudi Arabia; ^21^Department of Life Sciences and Public Health, Fondazione Policlinico Universitario A. Gemelli IRCCS, Rome, Italy; ^22^Department of Pediatrics, Antwerp University Hospital, Antwerp, Belgium; ^23^Center for Health Economics Research and Modelling Infectious Diseases (CHERMID), Vaccine and Infectious Disease Institute, University of Antwerp, Wilrijk, Belgium; ^24^Pediatric Rheumatology Unit, Department of Childhood and Developmental Medicine, Fatebenefratelli-Sacco Hospital, Milan, Italy; ^25^Clinical Pediatrics, Department of Molecular Medicine and Development, University of Siena, Siena, Italy; ^26^Rheumatology and Clinical Immunology Unit, Department of Internal Medicine, Faculty of Medicine, Cairo University, Giza, Egypt; ^27^Rheumatology Division, Hospital das Clinicas (HCFMUSP), Faculdade de Medicina, Universidade de São Paulo, São Paulo, Brazil; ^28^Joint Academic Rheumatology Program, Medical School, National and Kapodistrian University of Athens, Athens, Greece; ^29^Department of Internal Medicine, Pulmonology, Allergy and Clinical Immunology, Central Clinical Hospital of the Ministry of National Defence, Military Institute of Medicine, Warsaw, Poland; ^30^Vasculitis Research Unit, Autoinflammatory Diseases Clinical Unit, Department of Autoimmune Diseases, Hospital Clinic of Barcelona, August Pi i Sunyer Biomedical Research Institute (IDIBAPS), University of Barcelona, Barcelona, Spain; ^31^Rheumatology Unit, Department of Medicine, College of Medicine, King Saud University, Riyadh, Saudi Arabia; ^32^College of Medicine Research Center, College of Medicine, King Saud University, Riyadh, Saudi Arabia; ^33^Bioengineering and Biomedical Data Science Lab, Department of Medical Biotechnologies, University of Siena, Siena, Italy; ^34^Faculty of Medicine, Newgiza University, 6th of October City, Egypt; ^35^Ophthalmology Unit, Department of Medicine, Surgery and Neurosciences, University of Siena, Siena, Italy; ^36^Rare Diseases and Periodic Fevers Research Centre, Università Cattolica del Sacro Cuore, Rome, Italy

**Keywords:** PFAPA syndrome, autoinflammatory disease, International Registry, *Streptococcus salivarius* K12, probiotic, tonsillitis, prophylaxis

## Abstract

**Objective:**

To evaluate the potential role of *Streptococcus salivarius* K12 (SSK12) in controlling febrile flares in patients with Periodic Fever, Aphthous stomatitis, Pharyngitis, and cervical Adenitis (PFAPA) syndrome. Further aims were to assess the impact of SSK12 on (i) flare duration, (ii) variation in the degree of the highest body temperature during flares, (iii) steroid-sparing effect, and (iv) change of PFAPA accompanying symptoms before and after SSK12 introduction.

**Patients and methods:**

The medical charts from 85 pediatric patients with PFAPA syndrome (49 males and 36 females) enrolled in the AIDA registry and treated with SSK12 for a median period of 6.00 ± 7.00 months in the period between September 2017 and May 2022 were examined. Children recruited had a median time of disease duration of 19.00 ± 28.00 months.

**Results:**

The number of febrile flares significantly decreased comparing the 12 months before [median (IQR), 13.00 (6.00)] and after SSK12 initiation [median (IQR), 5.50 (8.00), *p* < 0.001]. The duration of fever was significantly reduced from 4.00 (2.00) days to 2.00 (2.00) days [*p* < 0.001]. Similarly, the highest temperature in°C was found significantly lower in the last follow-up assessment [median (IQR), 39.00 (1.00)] compared to the period prior to SSK12 start [median (IQR), 40.00 (1.00), *p* < 0.001]. Steroid load (mg/year) of betamethasone (or any equivalent steroid) significantly decreased between 12 months before treatment with SSK12 [median (IQR), 5.00 (8.00) mg/year] and the last follow-up visit [median (IQR), 2.00 (4.00) mg/year, *p* < 0.001]. The number of patients experiencing symptoms including pharyngitis/tonsillitis (*p* < 0.001), oral aphthae (*p* < 0.001) and cervical lymphadenopathy (*p* < 0.001) significantly decreased following SSK12.

**Conclusion:**

SSK12 prophylaxis given for at least 6.00 months was found to reduce febrile flares of PFAPA syndrome: in particular, it halved the total number per year of fever flares, shortened the duration of the single febrile episode, lowered body temperature by 1°C in the febrile flare, provided a steroid-sparing effect, and significantly reduced the accompanying symptoms related to the syndrome.

## Introduction

Periodic Fever, Aphthous stomatitis, Pharyngitis, and cervical Adenitis (PFAPA) syndrome represents the most common cause of periodic fever occurring in childhood (1). Recent findings proving a dysregulation in the machinery of innate immunity and a significant inflammasome-mediated activation during febrile flares have allowed PFAPA syndrome classification among multifactorial non-hereditary autoinflammatory disorders (2). The syndrome, initially described in 1987 (3), starts with fever attacks accompanied by at least one among three non-specific symptoms, which give name to the disease: aphthous stomatitis, pharyngitis, and cervical adenitis. PFAPA flares usually last 3-to-7 days and recur at intervals of 2–8 weeks, resulting highly predictable with “clockwork” regularity in most cases occurring in childhood (4). Clinical manifestations are separated by variable intervals of complete wellness without upper respiratory tract symptoms or evidence of chronic infections (5). Although children under the age of 5 constitute the largest number of patients, the disease has been recognized in older children, adolescents and even young adults (6). Despite a favorable outcome in most cases, patients’ quality of life is significantly impaired both in children and their caregivers (7). Treatment of the syndrome is usually symptomatic with a single low-dose betamethasone (0.1 mg/kg) or prednisone (1 mg/kg), being the most effective option to abort acute disease flares (8). Colchicine has also been proven to be effective in preventing PFAPA flares (9). Different reports have also revealed that tonsillectomy may provide symptom resolution in PFAPA syndrome, though the disease might reoccur later, at least in a minority of patients (10). Based on clinical efficacy and safety as a complementary treatment along with standard medical therapies in children with recurrent upper respiratory infections (11), the oropharyngeal probiotic *Streptococcus salivarius* K12 (SSK12) has been proposed as a tool to manage PFAPA syndrome. More in detail, it is established that SSK12 releases salivaricin A2 and B, two anti-bacterial molecules that antagonize the growth of *Streptococcus pyogenes*, the most important cause of pharyngeal bacterial infections in humans (12). The prophylactic administration of SSK12 to PFAPA patients might indeed counteract disease pathogenesis at the oral cavity level. Herein, we have examined a multi-center experience in pediatric PFAPA patients enrolled in the AutoInflammatory Disease Alliance (AIDA) International Registry treated with SSK12, focusing its impact on febrile attacks of such patients.

## Patients and methods

### Participants and study design

The cohort was selected among 233 patients with PFAPA syndrome enrolled in the AIDA International Registry, a physician-driven electronic-based platform designed for both retrospective and prospective collection of data. Solid evidence drawn from the collection of “real-life” standardized data represents the main goal of the Registry, that will hopefully lead to an overall improvement of the disease management (13). Patients with insufficient follow-up data or missing baseline values were excluded from the final cohort as well as patients with a follow-up shorter than 3 months. Data were collected both retrospectively and prospectively. The retrospective phase referred to real-world demographic, clinical, laboratory, instrumental and therapeutic data available at the time of enrollment into the Registry. Prospective data included clinical, therapeutic, and socio-economic information accrued during the subsequent follow-up visits. The medical records of 85 patients diagnosed with PFAPA syndrome according to Marshall criteria (3), modified by Thomas et al. (14), and according to Eurofever/PRINTO classification criteria (15), all treated with SSK12, were examined. SSK12 was given at the standard dose of 1 billion of colony-forming units/tablet of *Streptococcus salivarius* K12: the tablet was administered before night sleep, asking the patient to slowly dissolve it in the oral cavity, without biting or swallowing.

### Aims and endpoints

Primary aim of the study was to evaluate SSK12 efficacy on the recurrence of PFAPA febrile flares assessing any statistically significant difference between the number of PFAPA flares in the 12 months before SSK12 initiation and the last follow-up visit: for this specific analysis only patients with at least 12 months of follow-up were considered. Secondary aims were to evaluate SSK12 impact on: (i) febrile flare duration between 12 months before SSK12 initiation and the last follow-up visit, (ii) variation in the degree of the highest body temperature recorded in the 12 months before SSK12 treatment and the last follow-up, (iii) corticosteroid-sparing effect following SSK12 administration measured by betamethasone (or any glucocorticoid equivalent) load/year [specifically, the conversion to betamethasone for prednisone and methylprednisolone was done by multiplying doses by 0.16 and 0.20, respectively], and (iv) modification of PFAPA symptoms before and after SSK12 introduction.

### Protocol approval

The study protocol conformed to the tenets of the Declaration of Helsinki and received approval by the Ethics Committee of the University of Siena (Reference No. 14951). All centers participating in data collection of PFAPA patients received a formal approval by their local Ethics Committees. Informed consent was obtained from all participants, including patients’ parents or legal guardian/next of kin of each patient.

### Statistical analysis

Data were analyzed using IBMSPSS Statistics for Windows, version 28 (IBM Corp., Armonk, NY, United States). Descriptive statistics was employed to display mean and standard deviation (SD) or median and interquartile range (IQR), as required. Shapiro–Wilk test was used to assess the normality of data. Differences in means were investigated by the Mann–Whitney U test. Matched data were compared with Wilcoxon signed-rank test. McNemar test was used to analyze paired dichotomous variables. A *p* value lower than 0.05 was considered as statistically significant and all *p*-values were two-tailed.

## Results

**FIGURE 1:**
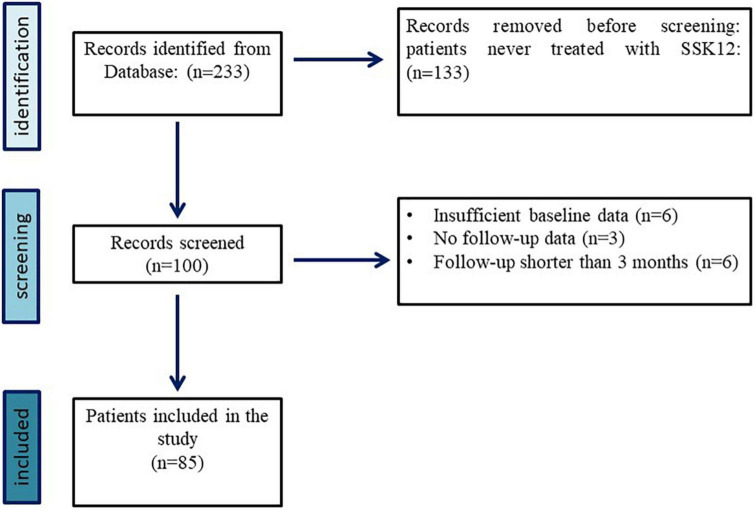
Selection process of eligible patients with PFAPA syndrome enrolled in the AIDA registry, forming the final cohort of participants in our study.

We examined the medical charts of 85 PFAPA patients (49 males, 36 females) receiving SSK12 in the period September 2017–May 2022. This cohort was selected among 233 PFAPA patients enrolled in the AIDA registry (13). Figure 1 shows the flow-diagram of the selection process of eligible patients, forming the final cohort of participants. Median (IQR) age of patients at SSK12 initiation was 4.58 (3.42) years. Demographic and therapeutic data have been summarized in Table 1. Tonsillectomy had been performed in only 1 patient prior to SSK12 initiation. Median (IQR) time of disease duration at SSK12 initiation was equal to 19.00 ± 28.00 months, and median (IQR) treatment duration with SSK12 was 6.00 ± 7.00 months. Genetic testing was performed in 22 patients: mostly it was assessed with Next Generation Sequencing (*n* = 11), followed by the search for specific mutations (*n* = 5), analysis of selected genes (*n* = 4), and complete gene sequencing (*n* = 2). Mutations were found in 6 patients: 3 variants being of unknown significance, 1 pathogenetic variant, 1 likely benign variant, and the remaining 1 still unclassified variant.

**TABLE 1:** Demographic and therapeutic data of the cohort of patients with PFAPA syndrome recruited from the AIDA International Registry and treated with SSK12.

Patients, *n*°	85
Males/females	49/36
Median (IQR) age at last follow-up visit (years)	4.58 (3.42)
Median (IQR) age at onset ± (months)	24.00 (22.50)
Median (IQR) age at SSK12 start (months)	55.00 (43.00)
Median follow-up ± IQR (months)	6.00 (7.00)
Median (IQR) disease duration before SSK12 treatment (months)	19.00 (28.00)
Previous treatments	GCs (*n* = 75) NSAIDs and/or acetaminophen (*n* = 51) Colchicine (*n* = 1)
Concomitant treatments	GCs (*n* = 71) NSAIDs and/or acetaminophen (*n* = 32)
Genetic data	Patients undergoing genetic testing (*n* = 22) Mutations encountered (*n* = 6): [*MEFV* (E148Q); *MEFV* (E230K); *MEFV* (V726A); *NLRP3* (c.2113C > A); *TLR9*; *ALPK1*]

ALPK1, Alpha-protein kinase 1; GCs, glucocorticoids; IQR, interquartile range; MEFV, Familial Mediterranean fever gene; NSAIDs, non-steroidal anti-inflammatory drugs; NLRP3, nucleotide-binding domain, Leucine-rich–containing family, Pyrin domain–containing-3; TLR9, toll-like receptor 9.

The number of febrile flares significantly decreased between the 12 months before SSK12 initiation [median (IQR), 13.00 (6.00)] and the last follow-up assessment [median (IQR), 5.50 (8.00), *p* < 0.001]. Seventy two out of 85 (84,7%) patients experienced a reduction in the number of febrile flares, with 66 of them also displaying a decrease in the duration of fever. Indeed, the duration of fever during flares was significantly reduced from 12 months prior to treatment with SSK12 [median (IQR), 4.00 (2.00) days] to the last follow-up visit [median (IQR), 2.00 (2.00) days, *p* < 0.001]. Similarly, the highest temperature recorded in each flare expressed in°C was found to be significantly lower in the last follow-up assessment [median (IQR), 39.00 (1.00)] compared to the period prior to SSK12 start [median (IQR), 40.00 (1.00), *p* < 0.001].

With regard to the steroid-sparing effect, the load (mg/year) of betamethasone (or any equivalent steroid) significantly decreased between 12 months before treatment with SSK12 [median (IQR), 5.00 (8.00) mg/year] and the last follow-up visit [median (IQR), 2.00 (4.00) mg/year, *p* < 0.001].

**FIGURE 2:**
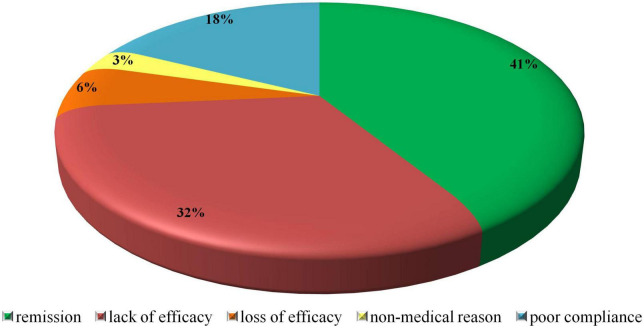
Reasons for SSK12 discontinuation in patients with PFAPA syndrome enrolled in the AIDA registry participating in the study.

The number of patients experiencing disease symptoms while on SSK12 treatment significantly decreased. Specifically, pharyngitis/tonsillitis (*p* < 0.001), oral aphthae (*p* < 0.001) and cervical lymphadenopathy (*p* < 0.001) were significantly reduced in the last follow-up visit. Also, other accompanying symptoms such as arthralgia (*p* < 0.001), abdominal pain (*p* < 0.001), and headache (*p* = 0.039) significantly decreased. Table 2 details the clinical PFAPA features of all patients participating in the study before SSK12 administration and the last follow-up assessment. No adverse events were detected during the overall treatment with SSK12. In addition, SSK12 was discontinued in 34 patients for the following reasons: remission (*n* = 14), lack of efficacy (*n* = 11), poor compliance (*n* = 6), loss of efficacy (*n* = 2), and non-medical reason (*n* = 1) (the pie chart in Figure 2 illustrates all reasons for SSK12 discontinuation). Patients discontinuing SSK12 treatment due to remission were characterized by a median (IQR) age at onset of 35 (27) months, a median (IQR) disease duration at SSK12 initiation of 8 (10) months, and a median (IQR) treatment duration of 6 (21) months.

**TABLE 2:** Clinical features of patients with PFAPA syndrome recruited from the AIDA International Registry in the 12-month period prior to SSK12 initiation and at the last follow-up assessment.

Clinical features	12 months before treatment with SSK12	Last follow-up visit	*p*-value
Headache	17	9	0.039
Pharyngitis/tonsillitis	80	50	<0.001
Oral aphthae	46	21	<0.001
Genital aphthae	0	0	Not performed
Skin manifestations	2	1	1.000
Cervical lymphadenopathy	68	38	<0.001
Hepatosplenomegaly	0	0	Not performed
Abdominal pain	23	8	<0.001
Vomiting	5	0	0.063
Diarrhea	4	1	0.250
Arthralgia/myalgia	32	15	<0.001
Arthritis	0	0	Not performed
Serositis	0	0	Not performed

## Discussion

Periodic Fever, Aphthous stomatitis, Pharyngitis, and cervical Adenitis syndrome is a complex multi-factorial autoinflammatory disorder characterized by glucocorticoid-responsive cyclic inflammatory episodes mainly occurring in the oral cavity (16). From a pathogenetic point of view, a host of studies have shown that the presence of SSK12 in the oral cavity hinders many bacteria and viruses implicated in pharyngotonsillitis and reduces the levels of several cytokines involved in the development of different inflammatory responses (17, 18). Furthermore, SSK12 produces bacteriocins which interfere with the growth of *Streptococcus pyogenes*, *Haemophilus influenzae*, *Streptococcus pneumoniae*, and *Moraxella catarrhalis*, commonly involved in the pathogenesis of oropharyngeal inflammation and/or acute otitis media (19). The hypothesis of a cyclic dysfunction of innate immunity originating in the oral cavity of PFAPA patients prompted the use of SSK12 in this peculiar form of recurrent fever defined by oropharyngeal inflammation. As hypothesized by recent small reports, the 3-month-administration of SSK12 seemed to reduce the cardinal PFAPA manifestations and decrease the need of administering symptomatic drugs during febrile flares (20, 21).

To the best of our knowledge, the present study depicts the first real-life experience with the highest number of PFAPA patients, assessing efficacy of SSK12 in the management of PFAPA syndrome. In this regard, we detected a significant reduction of the number of febrile flares between 12 months before and after SSK12 introduction. Additionally, our findings suggest a significant improvement in the features of each flare in terms of fever duration and maximal degree of body temperature recorded during flares. Specifically, flare duration was significantly reduced from 12 months prior to SSK12 treatment to the last follow-up assessment, showing a median reduction of 2 febrile days in each flare; we also found that patients displayed a 1°C reduction of the highest temperature recorded in each flare at the last follow-up visit compared to 12 months before SSK12 use.

Concerning the steroid-sparing effect, the yearly load of glucocorticoids significantly decreased after treatment with SSK12. A decreased intake of glucocorticoids might consent to avoid any eventual steroid-related side effects, which is of paramount importance particularly in the pediatric age. Finally, febrile episodes were less symptomatic and intense during treatment with SSK12. To this end, accompanying symptoms including pharyngitis/tonsillitis, oral aphthae and lymphoadenopathy were significantly reduced during febrile episodes, which is in agreement with previous small case reports describing the use of SSK12 in PFAPA patients (20, 21). Other probiotics such as *Lactobacillus plantarum* HEAL9 and *Lactobacillus paracasei* 8700:2 have been hypothesized to influence flare frequency in PFAPA patients (22). Interestingly, patients discontinuing treatment with SSK12 due to remission were found to have a shorter time between disease onset and SSK12 initiation, advocating for a better outcome in case of an early treatment. Indeed, the likelihood of treatment discontinuation due to remission might be higher in patients receiving SSK12 earlier. This hypothesis, however, deserves to be explored in further studies with larger samples and longer follow-up.

Several limitations should be considered. First of all, despite the considerable number of patients evaluated, a larger sample size of PFAPA patients would provide more robust evidence and shed light in additional topics by stratifying the cohort by several variables. For instance, the concomitant use of colchicine as a covariate is an interesting aspect worth investigating. Secondly, a longer follow-up is required to clarify other treatment aspects like the minimum exposure to SSK12 needed to obtain the maximum benefit in terms of febrile flares, duration of each flare or body temperature reduction and to verify predictors of drug-free remission. Thirdly, the lack of a control group is another issue that may limit the generalization of our findings. The lack of a control group also makes troublesome to assess the subgroup of patients undergoing a natural attenuation of the disease over time. However, it is reasonable to assume that the number of patients potentially experiencing a spontaneous resolution is relatively small since this event occurs mostly before puberty or in early adolescence (6, 23), while the cohort in our study was treated with SSK12 at a median age of 4.6 years. Finally, in the context of this multi-center collaborative study, the decision on when to start treatment with SSK12 was made by the local physician without any predefined standardized criteria. Of course, more in-depth investigational studies should disclose interesting results in this regard.

## Conclusion

In summary, our evaluation has found SSK12 to be effective in the reduction of febrile flares related to PFAPA syndrome in children. In particular, the administration of SSK12 as a prophylaxis tool for a median period of 6.00 has halved the total number per year of PFAPA flares, shortened the duration of the single fever flare, lowered body temperature by 1°C in the febrile episode, provided a steroid-sparing effect, and significantly reduced the accompanying symptoms related to the syndrome. Altogether, these findings suggest SSK12 as a promising treatment alternative in the management of PFAPA syndrome. Further investigations are needed to corroborate these preliminary data and establish the right place of this probiotic in the treatment algorithm of PFAPA syndrome with the ultimate goal to optimize and personalize the management in accordance with clinical patient’s profile.

## Data availability statement

The raw data supporting the conclusions of this article will be made available by the authors, upon reasonable request.

## Ethics statement

The studies involving human participants were reviewed and approved by Ethics Committee of Azienda Ospedaliera Universitaria Senese, Siena, Italy. Written informed consent to participate in this study was provided by the participants’ legal guardian/next of kin.

## Author contributions

FL and JS wrote the first draft of the manuscript. DR and FL conceived and designed the study. DR supervised the project. LC conceived and designed the study and accounts for AIDA Registries Coordinator. AB was the bioengineer involved in the technical management of the platform and registries. FL, AI, GC, ED, RLu, LB, MM, AC, VM, RLo, MN, CC, MR, SP, CGe, AV, VC, CGa, FD, MC, GR, GE, PP, SG, FCaru, EA, BOz, FS, AcM, PB, CM, FCard, LI, BOg, AnM, MT, AA-MAM, BF, and DR were involved in data recruitment in the Registry dedicated to patients with PFAPA syndrome. GL, AT, IA, PS, KL, EW-S, JH-R, VG-C, CF, and LC were included in the authorship as investigators from the top contributor centers for any of the other eight AIDA Registries; authorship has been established based on the number of data recruited in the AIDA Network Registries on 4 November 2022. All authors contributed to the article and approved the submitted version.

## References

[1] Feder H Salazar JC. A clinical review of 105 patients with PFAPA (a periodic fever syndrome). *Acta Paediatr.* (2010) 99:178–84. 10.1111/j.1651-2227.2009.01554.x 19889105

[2] Rigante D Frediani B Galeazzi M Cantarini L. From the Mediterranean to the sea of Japan: the transcontinental odyssey of autoinflammatory diseases. *Biomed Res Int.* (2013) 2013:485103. 10.1155/2013/485103 23971037 PMC3736491

[3] Marshall G Edwards K Butler J Lawton A. Syndrome of periodic fever, pharyngitis, and aphthous stomatitis. *J Pediatr.* (1987) 110:43–6. 10.1016/s0022-3476(87)80285-8 3794885

[4] Wang A Manthiram K Dedeoglu F Licameli G. Periodic fever, aphthous stomatitis, pharyngitis, and adenitis (PFAPA) syndrome: a review. *World J Otorhinolaryngol Head Neck Surg.* (2021) 7:166–73. 10.1016/j.wjorl.2021.05.004 34430824 PMC8356195

[5] Gentileschi S Vitale A Frediani B Galeazzi M Rigante D Cantarini L. Challenges and new horizons in the periodic fever, aphthous stomatitis, pharyngitis and adenitis (PFAPA) syndrome. *Expert Opin Orphan Drugs.* (2017) 5:165–71. 10.1080/21678707.2017.1279049

[6] Gaggiano C Rigante D Sota J Grosso S Cantarini L. Treatment options for periodic fever, aphthous stomatitis, pharyngitis, and cervical adenitis (PFAPA) syndrome in children and adults: a narrative review. *Clin Rheumatol.* (2019) 38:11–7. 10.1007/s10067-018-4361-2 30488366

[7] Grimwood C Koné-Paut I Piram M Rossi-Semerano L Hentgen V. Health-related quality of life in children with PFAPA syndrome. *Orphanet J Rare Dis.* (2018) 13:132. 10.1186/s13023-018-0878-3 30092788 PMC6085641

[8] Rigante D Corina L. Periodic fever, aphthous stomatitis, pharyngitis and cervical adenitis (PFAPA) syndrome: a debate about diagnosis and treatment in children continues. *Int J Pediatr Otorhinolaryngol.* (2020) 130:109830. 10.1016/j.ijporl.2019.109830 31866107

[9] Raeeskarami S Sadeghi P Vahedi M Asna Ashari K Mousavi TM Ziaee V. *Colchicine versus* cimetidine: the better choice for Periodic fever, aphthous stomatitis, pharyngitis, adenitis (PFAPA) syndrome prophylaxis, and the role of *MEFV* gene mutations. *Pediatr Rheumatol Online J.* (2022) 20:72. 10.1186/s12969-022-00733-3 36045426 PMC9428878

[10] Gozen ED Yildiz M Kara S Tevetoglu F Haslak F Adrovic A Long-term efficacy of tonsillectomy/adenotonsillectomy in patients with periodic fever aphthous stomatitis pharyngitis adenitis syndrome with special emphasis on co-existence of familial Mediterranean fever. *Rheumatol Int.* (2022) 43:137–45. 10.1007/s00296-022-05210-4 36116090

[11] Guo H Xiang X Lin X Wang Q Qin S Lu X Oropharyngeal probiotic ENT-K12 as an effective dietary intervention for children with recurrent respiratory tract infections during cold season. *Front Nutr.* (2022) 9:900448. 10.3389/fnut.2022.900448 35634421 PMC9132010

[12] Wescombe P Hale J Heng N Tagg J. Developing oral probiotics from *Streptococcus salivarius*. *Future Microbiol.* (2012) 7:1355–71. 10.2217/fmb.12.113 23231486

[13] Della Casa F Vitale A Cattalini M La Torre F Capozio G Del Giudice E Development and implementation of the AIDA International Registry for patients with Periodic Fever, Aphthous stomatitis, Pharyngitis, and cervical Adenitis syndrome. *Front Pediatr.* (2022) 10:930305. 10.3389/fped.2022.930305 35935379 PMC9353299

[14] Thomas K Feder H Lawton A Edwards K. Periodic fever syndrome in children. *J Pediatr.* (1999) 135:15–21. 10.1016/s0022-3476(99)70321-5 10393598

[15] Gattorno M Hofer M Federici S Vanoni F Bovis F Aksentijevich I Classification criteria for autoinflammatory recurrent fevers. *Ann Rheum Dis.* (2019) 78:1025–32. 10.1136/annrheumdis-2019-215048 31018962

[16] Sangiorgi E Rigante D. The clinical chameleon of autoinflammatory diseases in children. *Cells.* (2022) 11:2231. 10.3390/cells11142231 35883675 PMC9318468

[17] Gregori G Righi O Risso P Boiardi G Demuru G Ferzetti A Reduction of group A beta-hemolytic streptococcus pharyngo-tonsillar infections associated with use of the oral probiotic *Streptococcus salivarius* K12: a retrospective observational study. *Ther Clin Risk Manag.* (2016) 12:87–92. 10.2147/TCRM.S96134 26855579 PMC4725641

[18] Di Pierro F Colombo M Zanvit A Risso P Rottoli A. Use of *Streptococcus salivarius* K12 in the prevention of streptococcal and viral pharyngo-tonsillitis in children. *Drug Healthc Patient Saf.* (2014) 6:15–20. 24600248 10.2147/DHPS.S59665 PMC3928062

[19] Hyink O Wescombe PA Upton M Ragland N Burton JP Tagg JR. Salivaricin A2 and the novel lantibiotic salivaricin B are encoded at adjacent loci on a 190-kilobase transmissible megaplasmid in the oral probiotic strain *Streptococcus salivarius* K12. *Appl Environ Microbiol.* (2007) 73:1107–13. 10.1128/AEM.02265-06 17194838 PMC1828679

[20] Di Pierro F Donato G Fomia F Adami T Careddu D Cassandro C Preliminary pediatric clinical evaluation of the oral probiotic *Streptococcus salivarius* K12 in preventing recurrent pharyngitis and/or tonsillitis caused by *Streptococcus pyogenes* and recurrent acute otitis media. *Int J Gen Med.* (2012) 5:991–7. 23233809 10.2147/IJGM.S38859 PMC3516470

[21] Di Pierro F Campana A Panatta M Antenucci V De Vincentiis G. The use of *Streptococcus salivarius* K12 in attenuating PFAPA syndrome, a pilot study. *Altern Integr Med.* (2016) 5:4. 10.4172/2327-5162.1000222

[22] Batu ED Kaya Akca U Basaran O Bilginer Y Özen S. Probiotic use in the prophylaxis of periodic fever, aphthous stomatitis, pharyngitis, and adenitis (PFAPA) syndrome: a retrospective cohort study. *Rheumatol Int.* (2022) 42:1207–11. 10.1007/s00296-021-05084-y 34994815

[23] Vanoni F Theodoropoulou K Hofer M. PFAPA syndrome: a review on treatment and outcome. *Pediatr Rheumatol Online J.* (2016) 14:38. 10.1186/s12969-016-0101-9 27349388 PMC4924332

